# Epidermal Growth Factor Receptor and Ki-67 as Predictive Biomarkers Identify Patients Who Will Be More Sensitive to Intravesical Instillations for the Prevention of Bladder Cancer Recurrence after Radical Nephroureterectomy

**DOI:** 10.1371/journal.pone.0166884

**Published:** 2016-11-21

**Authors:** Xingbo Long, Xiongbing Zu, Yuan Li, Wei He, Xiheng Hu, Shiyu Tong, Zhi Wang, Minfeng Chen, Lin Qi

**Affiliations:** Department of Urology, Xiangya Hospital, Central South University, Changsha, Hunan, China; National Institute of Health, UNITED STATES

## Abstract

**Background:**

To date, prophylactic intravesical chemotherapy after radical nephroureterectomy is one of the few available treatments that effectively prevent secondary bladder cancer. However, treating all patients with prophylactic intravesical chemotherapy is excessive for patients who are at a low risk or insensitive to the treatment. Thus, to guide individualized clinical treatment, in addition to identifying patients who are at risk of bladder cancer recurrence, it is equally necessary to identify the patients who will benefit the most from prophylactic, postoperative intravesical instillation therapy.

**Methods:**

Epidermal growth factor receptor (EGFR) and Ki-67 expression levels were measured using immunohistochemical staining samples from 320 patients with upper urinary tract urothelial carcinoma (UTUC) from 2004 to 2012. Although no patients received intravesical chemotherapy after RNU before 2008, this method began to be used in 2008 to prevent bladder cancer recurrence. To identify the patients who would most benefit from intravesical chemotherapy, we assessed biological interactions between intravesical chemotherapy and clinicopathological factors or biomarkers.

**Results:**

The incidence rates of bladder UTUC recurrence decreased after intravesical chemotherapy, and the decrease was greater in patients with low Ki-67 levels, negative EGFR staining and preoperative positive urine cytology. Biological interactions were observed between intravesical chemotherapy, low-level Ki-67 and EGFR negativity. The multivariate analysis showed that after balancing a variety of factors, intravesical chemotherapy is a protective factor for preventing intravesical recurrence in the negative EGFR, low-level Ki-67 and preoperative positive urine cytology sub-groups but not in their corresponding sub-groups. Additionally, the multivariate analysis revealed that preoperative positive urine cytology and Ki-67 were not but that EGFR positivity was an independent risk factor for recurrence after intravesical chemotherapy.

**Conclusions:**

Patients with low Ki-67 levels, negative EGFR staining and preoperative positive urine cytology appear to be more sensitive to intravesical instillations for bladder recurrence prevention after RNU.

## Introduction

Upper urinary tract urothelial carcinoma (UTUC) is rare and accounts for only 5–10% of urothelial carcinoma [1]. The gold standard treatment for localized UTUC is radical nephroureterectomy (RNU) and excision of the bladder cuff [2]; however, approximately 20–50% of patients will experience bladder cancer recurrence after RNU [1]. To date, prophylactic intravesical chemotherapy after RNU is one of the few available treatments that effectively prevent secondary bladder cancer [3,4]; however, treating all patients with prophylactic intravesical chemotherapy would be excessive for those who are low risk or insensitive to the treatment. Thus, to guide individualized clinical treatment, in addition to identifying patients who are at risk of bladder cancer recurrence, it is equally necessary to identify patients who will benefit the most from prophylactic, postoperative intravesical instillation therapy.

To our knowledge, although an increasing number of risk factors for bladder cancer recurrence after RNU has been recognized by previous studies [5], most factors were clinical and pathological characteristics. Few validated biomarkers have been effectively proven to predict prognosis and subsequent bladder recurrence. Based on previous studies, the proliferation markers Ki67 [6,7] and growth factor receptors EGFR [8,9] are two of a few biomarkers determined to have prognostic value for both bladder urothelial carcinoma and upper urinary tract urothelial carcinoma. Additionally, the expression of Ki67 and EGFR were important in predicting primary superficial bladder cancer recurrence and secondary bladder cancer after RNU [10–13]. However, though Ki67 and EGFR have the ability to predict bladder recurrence of the upper urinary tract urothelial carcinoma after RNU, the ability to predict which group of patients would be more sensitive to intravesical therapy remains unknown. The present study is the first to evaluate the role of the molecular markers EGFR and Ki-67 as well as conventional clinicopathological factors as predictors not only of bladder tumor recurrence after RNU in patients with and without intravesical chemotherapy but also of patients who will benefit the most from prophylactic, postoperative intravesical instillation therapy.

## Materials and Methods

This study was approved by our Medical Ethics Committee of Xiangya Hospital, Central South University. The need for informed consent was waived by our Medical Ethics Committee because the study was an observational, retrospective study using a database from which the patients’ identification information had been removed.

We retrospectively reviewed the clinical and histopathological records of patients with unilateral transitional cell carcinoma of the urinary tract who had undergone RNU at a large urology center in South Central China between January 1, 2004 and July 30 2012. Patients were excluded if they had clinical evidence of distant metastases, a follow-up of less than 12 months and no subsequent bladder cancer recurrence, neo-adjuvant chemotherapy, serious complications or other active neoplasms. To minimize the influence of primary bladder cancer recurrence, patients with concomitant or previous bladder tumors were also excluded from our study. In all, 320 qualified patients were enrolled in our study, and their major clinical characteristics are illustrated in Table 1.

**Table 1:** Patient and Tumor Characteristics Stratified by Group.

	Total (n = 320)	Group		Pv
		Group 1 (n = 159)	Group 2 (n = 161)	
Gender^†^				0.53
Male	233	113	120	
Female	87	46	41	
Age (years, mean ± SD) ^‡^	61.4±8.3	62.0±8.0	60.8±8.6	0.19
Tumor location^†^				1.0
Calix or pelvis	213	106	107	
Ureter	107	53	54	
Multifocality^†^				0.20
Solitary	240	114	126	
Multiple	80	45	35	
Preoperative urine cytology^†^				0.50
Positive	139	66	73	
Negative	181	93	88	
Laterality^†^				0.58
Right	157	81	76	
Left	163	78	85	
Grade^†^				0.81
Grade 1, 2	229	115	114	
Grade 3	91	44	47	
Pathological T stage^§^				0.62
PT1 or less	109	50	59	
PT2	97	50	47	
PT3 or more	114	59	55	
Surgical approach^†^				<0.001*
Open	68	49	19	
Laparoscopic	252	110	142	
Management of distal ureter^†^				0.11
Bladder cuff excision	300	153	147	
Pluck technique	20	6	14	
Adjuvant chemotherapies^†^				0.32
Present	42	24	18	
Absent	278	135	143	
BMI (kg/m^2^, mean ± SD)^‡^	22.5±1.5	22.4±1.5	22.6±1.5	0.20
EGFR^†^				0.74
Positive	136	66	70	
Negative	184	93	91	
Ki-67^†^				0.51
Positive	165	85	80	
Negative	155	74	81	
Status of surgical margins(positive) ^†^				1.0
Positive	10	5	5	
Negative	310	154	156	

†: Fisher's exact test (two-sided).

‡: Two-sided t-test, with equal variances assumed under Leven's test. The results are the mean±SD.

§: Wilcoxon rank sum test.

*: Statistically significant at P<0.05.

Before 2008, no patients received intravesical chemotherapy after RNU. However, to prevent bladder cancer recurrence after RNU, in January 2008, we started to use a single postoperative dose of intravesical chemotherapy (30 mg of epirubicin, retained for at least 30 min) on the day of catheter removal after RNU (approximately 1 to 2 weeks) as a standard treatment for all patients. The patients who received the procedure were provided with detailed information about the possible outcomes and risks of intravesical chemotherapy and had agreed to the procedure by signing a consent form beforehand. In all, 159 patients who did not receive intravesical chemotherapy after RNU were placed in group 1, and 161 patients who did receive intravesical chemotherapy were placed in group 2.

The first follow-up was performed 1 month after surgery. Then, cystoscopy, urinalysis, urine cytology and abdominal ultrasound results were evaluated every 3 months for the first 2 years and every 6 months thereafter; computerized tomography was performed every 6 months for the first 2 years and once per year thereafter.

During cystoscopy, all visible lesions were biopsied or resected, and bladder cancer recurrence was established only by histological evidence. The interval time was defined as the time between the date of RNU and the time when the first bladder tumor recurrence was detected. Tumor multiplicity was defined as the synchronous presence of two or more pathologically confirmed tumors in discontinuous locations. Tumor location was defined as either the renal pelvis or ureter; if multiple tumors existed in both the renal pelvis and ureter, the location was classified as the ureter. There are two methods to manage the distal ureter: open bladder cuff excision and the pluck technique. Tumors were graded histologically in accordance with the WHO three-tiered classification at the time of the initial diagnosis [14]. Clinical and pathological staging was performed according to the 2002 TNM classification [15].

For further analysis, the resected tissues of subsequently recurring bladder tumors were also examined using immunohistochemistry. The tissues of subsequently recurring bladder tumors from patients without prophylactic intravesical chemotherapy (before the year 2008) were classified as group A, and those from patients with prophylactic intravesical chemotherapy were classified as group B.

### Immunohistochemistry

The tissue microarray (TMA) technique described in detail by Kononen et al. [16] was used for immunohistochemical staining for the molecular markers Ki-67 and EGFR. In consideration of tumor heterogeneity, at least three cylindrical core biopsies were taken from different sites in each tumor and arrayed in a recipient paraffin TMA block.

Immunohistochemical staining for EGFR and Ki-67 was performed on 4-μm-thick TMA sections using an EGFR pharmDX® Kit (DakoCytomation, Glostrup, Denmark) with standardized, automated procedures (Dako Autostainer) and using mouse monoclonal antibodies (clone MIB-1, DAKO, Hamburg, Germany, dilution 1:50), respectively; these standard staining procedures were previously described [9,17,18]. The negative and positive controls for EGFR were cell lines provided by the manufacturer and run in each sample. The negative controls for Ki-67 were obtained by omitting the primary antibody.

**Fig 1:**
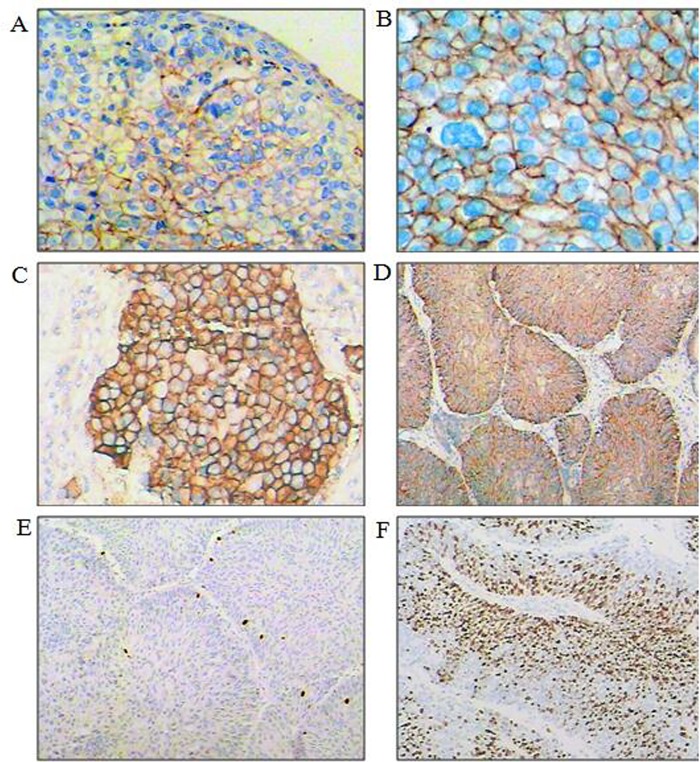
EGFR and Ki-67 Immunohistochemistry. (A) Relatively weak and incomplete membranous EGFR 1+ staining (magnification ×200) in a urothelial carcinoma (UC). (B) Moderate and mostly complete membranous EGFR 2+ staining (magnification ×400) in a UC. (C) Strong and complete membranous EGFR 3+ staining in a UC (magnification ×200). (D) Strong and complete membranous EGFR 3+ staining in a UC (magnification ×100). (E) Very low-level Ki-67 expression in a UC with few positive cells (magnification ×100). (F) Ki-67 overexpression in a UC with more than 50% positive cells (magnification ×100).

Immunoreactivity was independently assessed by two investigators (ZW and JBC) who were blinded to the clinicopathological data. In cases of discrepant results, the values were discussed until an agreement was reached. According to the EGFR pharmDX® Kit guidelines, membranous staining was required for a positive EGFR score. At least two cores of stained cancer tissue per case were considered necessary for the reliable evaluation of EGFR immunoreactivity. Following the recommendations for the interpretation of HER-2 immunostaining, the highest intensity of membrane staining was assessed as faintly perceptible (1+), moderately perceptible and complete (2+), or strongly perceptible and complete (3+); membrane staining stronger than 1+ was arbitrarily considered EGFR-positive (Fig 1A, 1B, 1C and 1D). The Ki-67 labeling index was defined as high or low if the nuclear staining of tumor cells was >15% or ≤15%, respectively (Fig 1E and 1F).

### Statistical Analyses

The cumulative incidence method and Gray’s test [19] were used to compare bladder tumor recurrence rates between two groups. To eliminate bias from Cox proportional hazards regression, the competing risks regression model of Fine and Gray was used for the univariate and multivariate analyses [19]. We used two sets of sloped lines to demonstrate the differences in the incidence rates of bladder recurrence between patients with and without certain biomarkers or conventional clinicopathological factors before or after intravesical chemotherapy to describe changes in the incidence rates of bladder recurrence 12, 18, 24, and 36 months before or after intravesical chemotherapy and to distinguish the regression coefficients between the two sets of lines. The method described by Andersson et al. [20] for measuring biological interactions was used to determine any biological interactions between intravesical chemotherapy and clinicopathological factors or biomarkers. In this calculation, two indexes, i.e., the attributable proportion due to interaction (AP) and the synergy index (S), are used to evaluate biological interactions. If significant biological interactions exist, the 95% confidence interval of AP should not include 0 and the 95% confidence interval of S should not include 1. The statistical tests were two-sided, and a P value <0.05 was considered statistically significant. Data were analyzed using R 2.13.0 (The R Foundation for Statistical Computing 2011, Vienna, Austria).

## Results

**Fig 2:**
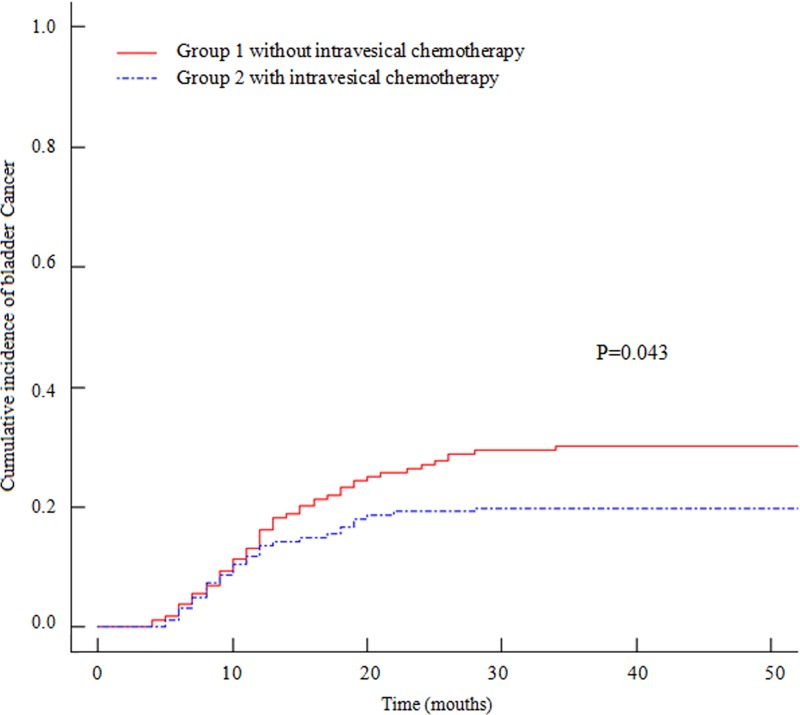
Cumulative Incidence Plot of Tumor Recurrence in the Bladder According to Groups. The cumulative incidence rates at 1 and 3 years after radical nephroureterectomy were 16.4% and 30.2%, respectively, in group 1 and 13.7% and 19.9%, respectively, in group 2 (P = 0.043).

The median (interquartile range) follow-up was 52 (38–68) months. In all, 129 of 320 (40.3%) patients died, and 80 (25.0%) developed subsequent bladder tumors at a median interval of 11.4 months (range 4–34) during the follow-up period. Significant differences were observed in the cumulative incidence rates of UTUC recurrence in the bladder at 1 and 3 years between group 1 (16.4% and 30.2%, respectively) and group 2 (13.7% and 19.9%, respectively) (Fig 2, P = 0.043).

### Interaction Analyses

In the sub-group analysis, we investigated interactions between intravesical chemotherapy and clinicopathological factors or biomarkers to determine whether the change of incidence rates of bladder recurrence after intravesical chemotherapy differed by patient group.

**Fig 3:**
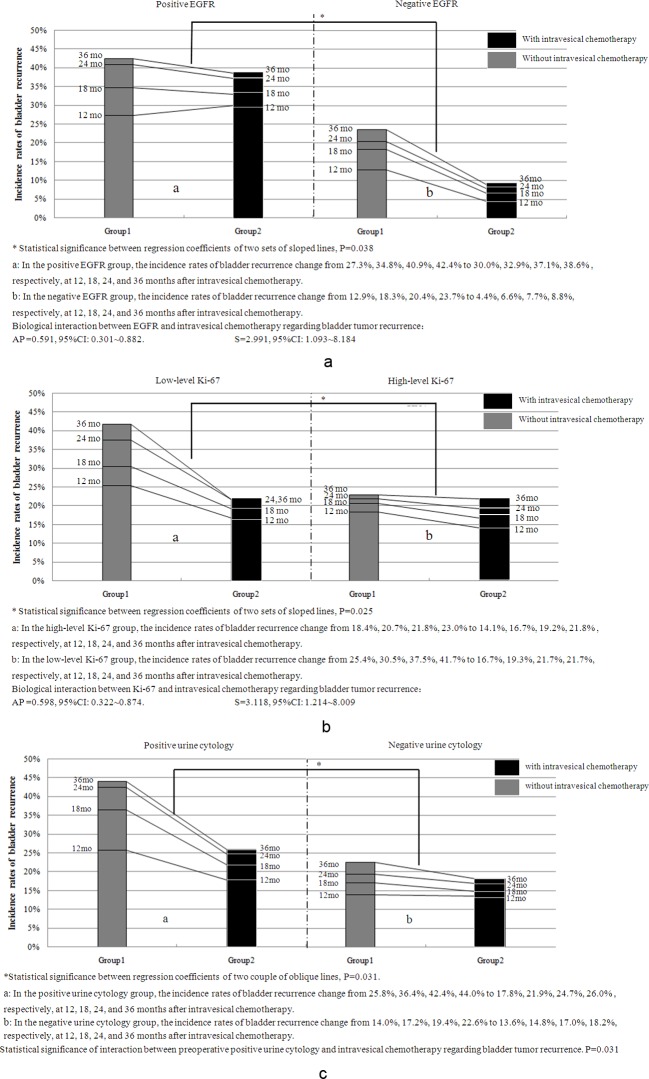
Differences in the Incidence Rates of Bladder Recurrence between Patients with and without Certain Biomarkers or Conventional Clinicopathological Factors Before or After Intravesical Chemotherapy. After intravesical chemotherapy, the incidence rates of bladder tumor recurrence decreased more in the EGFR-negative (A), low-level Ki-67 (B), and preoperative positive urine cytology (C) sub-groups than those in the corresponding sub-groups when comparing the regression coefficients of the two sets of oblique lines. A significant synergistic biological interaction was observed between intravesical chemotherapy and EGFR negativity (a, S = 2.991, AP = 0.591) or low-level Ki-67 expression (b, S = 3.118, AP = 0.598); however, no biological interaction was observed between preoperative urine cytology and intravesical chemotherapy.

We found that the incidence rates of UTUC recurrence in the bladder decreased more in the negative EGFR groups than in the positive EGFR groups after intravesical chemotherapy (Fig 3A, P = 0.038). Moreover, a significant synergistic biological interaction was observed between EGFR negativity and intravesical chemotherapy in preventing bladder tumor recurrence (Fig 3A, S = 2.991, AP = 0.591).

Additionally, after intravesical chemotherapy, the incidence rates of UTUC recurrence in the bladder decreased more in the low-level Ki-67 sub-groups than in the high-level Ki-67 sub-groups (Fig 3B, P = 0.025), and a synergistic biological interaction was observed between low-level Ki-67 labeling and without intravesical chemotherapy in leading to bladder tumor recurrence (Fig 3B, S = 3.118, AP = 0.598).

The cumulative incidence rates of UTUC recurrence in the bladder decreased more in the preoperative positive than the negative urine cytology group after intravesical chemotherapy (Fig 3C, P = 0.031). Although no biological interactions were observed between preoperative positive or negative urine cytology and with or without intravesical chemotherapy when using the method described by Andersson et al. [20], binary logistical regression analyses show that a significant interaction could be found between preoperative positive urine cytology and intravesical chemotherapy in regard to bladder tumor recurrence (Fig 3C, P = 0.031).

Additionally, the multivariate analyses on sub-groups revealed that after balancing varieties of factors, intravesical chemotherapy was a protective factor in preventing intravesical recurrence in negative EGFR, low-level Ki-67 and preoperative positive urine cytology sub-groups but not in the corresponding sub-groups (S1 Table).

With the exception of the three factors noted above, no statistical differences were observed when comparing the regression coefficients of decreased recurrence incidence rates after intravesical chemotherapy between patients with or without certain clinicopathological factors, such as ureteral tumor location and tumor multifocality. Furthermore, no interactions were observed between these clinicopathological factors and intravesical chemotherapy (S1 Fig).

### The change of bladder recurrence incidence rate after intravesical chemotherapy considering combinations of three factors

**Fig 4:**
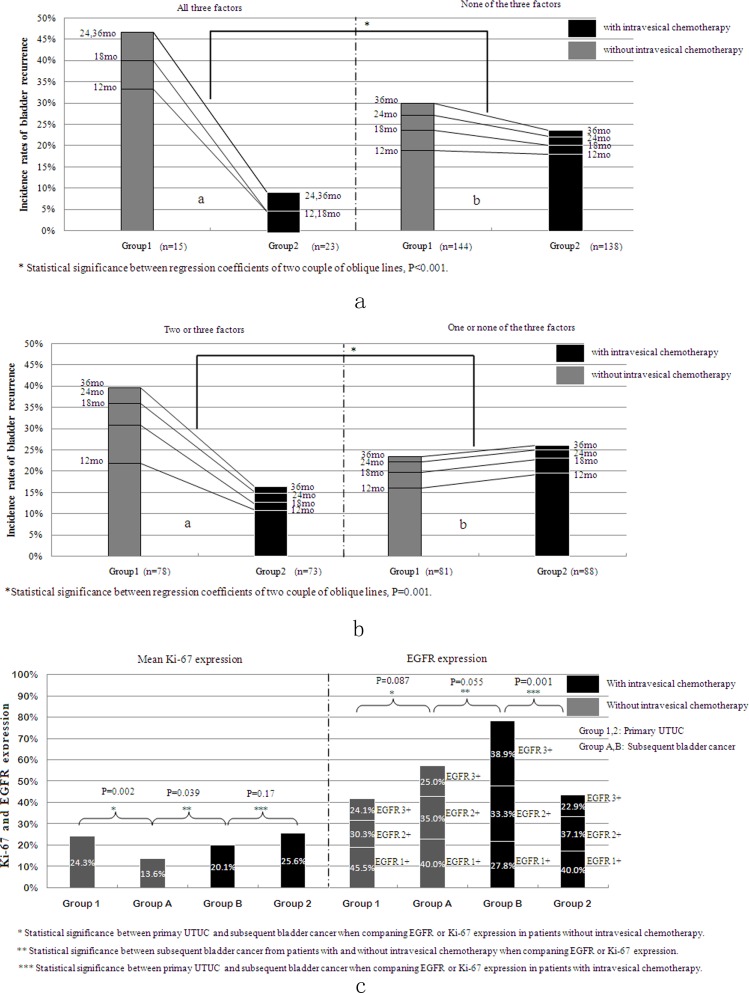
**(A, B) The Change in Bladder Recurrence Incidence Rate After Intravesical Chemotherapy Considering Combinations of Three Factors.** A) After intravesical chemotherapy, the rates of bladder tumor recurrence decreased more in groups exhibiting all three factors than in the corresponding sub-groups (P<0.001). B) After intravesical chemotherapy, the rates of bladder tumor recurrence decreased more in groups with either two or three factors than in the corresponding sub-groups (P = 0.001). C) A comparison of biomarker expression in recurrent bladder tumors between patients with and without prophylactic intravesical chemotherapy and between primary UTUC and recurrent bladder tumors.

We grouped patients according to three factors: negative EGFR staining, low-level Ki-67 staining and preoperative positive urine cytology. We then compared the changes in bladder tumor recurrence incidence rates after intravesical chemotherapy in patients with or without all three factors and in patients with zero to one or two to three factors. In patients with two or three factors, the bladder tumor recurrence rates decreased significantly more after intravesical chemotherapy than those in the corresponding sub-groups (Fig 4A, P<0.001; Fig 4B, P = 0.001).

### EGFR and Ki-67 expressions in recurrent bladder tumor

Operations for recurrent bladder cancer were performed in our hospital in 35 of 49 (71.4%) bladder recurrent patients in group 1 (group A) and 23 out of 31 (74.2%) bladder recurrent patients in group 2 (group B). Fig 4C shows the expression levels of Ki-67 and EGFR in the primary UTUC and the subsequent bladder cancer. We found that recurrent bladder tumors in patients with prophylactic intravesical chemotherapy expressed higher EGFR and Ki-67 levels than those in patients without prophylactic intravesical chemotherapy (Fig 4C, Group A vs Group B, P = 0.055, P = 0.039). In patients without prophylactic intravesical chemotherapy, the subsequently recurring bladder tumors expressed lower Ki-67 levels than those of primary the UTUC (Fig 4C, Ki-67: Group 1 vs Group A, P = 0.002), while in patients with prophylactic intravesical chemotherapy, the subsequently recurring bladder tumors expressed higher EGFR levels than those of primary the UTUC (Fig 4C, EGFR: Group 2 vs Group B, P = 0.001).

### The associations between Ki-67 / EGFR expression in the primary UTUC and clinicopathological parameters

High Ki-67 expression levels and EGFR overexpression were observed in 165 (51.6%) and 136 (42.5%) patients, respectively. Table 2 shows that Ki-67 overexpression was associated with only high tumor grade (P = 0.003), while EGFR overexpression was associated with multiple tumors (P = 0.050) and high tumor grade (P<0.001).

**Table 2:** Association between EGFR / Ki-67 Expression and Clinicopathological Parameters in 320 Patients.

	Ki-67 expression			EGFR expression		
	Low level(n = 155)	High level(n = 165)	Pv	Negative(n = 184)	Positive(n = 136)	Pv
Gender			0.80			1.0
Male	114	119		134	9	
Female	41	46		50	37	
Age (years, mean ± SD)	61.6±8.6	61.2±8.1	0.66	61.6±8.5	61.2±8.1	0.64
Tumor location			0.41			0.72
Calix or pelvis	107	106		124	89	
Ureter	48	59		60	47	
Multifocality			0.30			0.05*
Solitary	112	128		146	94	
Multiple	43	37		38	42	
Preoperative urine cytology			0.50			0.43
Positive	64	75		76	63	
Negative	91	90		108	73	
Laterality			0.31			0.26
Right	81	76		85	72	
Left	74	89		99	64	
Grade			0.003*			<0.001*
Grade 1, 2	123	106		47	82	
Grade 3	32	59		137	54	
Pathological T stage			0.17			0.95
PT1 or less	45	64		64	45	
PT2	49	48		55	42	
PT3 or more	61	53		65	49	
BMI (kg/m^2^, mean ± SD)	22.4±1.6	22.6±1.4	0.18	22.6±1.6	22.4±1.4	0.17
EGFR			0.26			—
Positive	71	65		—	—	
Negative	84	100		—	—	
Ki-67			—			0.26
Positive	—	—		100	65	
Negative	—	—		84	71	

* Statistically significant.

The results of the univariate and multivariate analyses in both groups 1 and group 2 are illustrated in S2 Table. The multivariate analysis revealed that while preoperative positive urine cytology and Ki-67 were not, EGFR positivity was an independent risk factor for recurrence after intravesical chemotherapy (group 2).

## Discussion

In our study, the bladder tumor recurrence rate decreased after prophylactic, postoperative intravesical instillation therapy. Further analysis showed that the recurrence rate decreased less in EGFR-positive patients than in EGFR-negative patients. Furthermore, there was a synergistic biological interaction between EGFR negativity and intravesical chemotherapy in preventing bladder recurrence, indicating that EGFR-positive patients may not be as sensitive to intravesical chemotherapy as EGFR-negative patients. Additionally, low-level Ki-67 patients appeared to be more sensitive to intravesical chemotherapy than high-level Ki-67 patients. The bladder recurrence rate after intravesical chemotherapy decreased more in low-level Ki-67 patients than in high-level Ki-67 patients, and a synergistic biological interaction could be observed between low-level Ki-67 staining and without intravesical chemotherapy in leading to bladder recurrence.

The bladder recurrence rate decreased more in patients with preoperative positive urine cytology than in those with preoperative negative urine cytology. A significant interaction was found between preoperative positive urine cytology and intravesical chemotherapy in regard to bladder tumor recurrence, indicating that patients with preoperative positive urine cytology may also be sensitive to intravesical chemotherapy.

Additionally, the multivariate analyses on different sub-groups revealed that after balancing varieties of factors, intravesical chemotherapy was a protective factor for preventing intravesical recurrence in the negative EGFR, low-level Ki-67 and preoperative positive urine cytology sub-groups; however, in the positive EGFR, high-level Ki-67 and preoperative negative urine cytology sub-groups, intravesical chemotherapy did not show a protective effect, which further confirmed that patients with positive EGFR, high-level Ki-67 and preoperative negative urine cytology may be less sensitive to intravesical chemotherapy than the corresponding sub-groups.

To date, two hypotheses explaining bladder tumor recurrence after nephroureterectomy have been proposed [21–23]: intraluminal cancer cell seeding and implanting and field cancerization. Although the seeding hypothesis has been supported by an increasing number of molecular studies [21,23,24], and by clinical evidence ^4^, both mechanisms may coexist and contribute to subsequent bladder tumor recurrence.

Intravesical chemotherapy is believed to effectively prevent the implantation (dispersion) of tumor cells into the bladder epithelium from another site and prevent implanted cells [25], as well as co-existing microscopic lesions, from establishing new tumors [4]. In other words, prophylactic intravesical chemotherapy after RNU can effectively decrease bladder tumor recurrence after RNU caused by intraluminal seeding. However, intravesical chemotherapy may have little effect on the occurrence of new, multifocal urothelial tumors that are metachronous in nature and could be explained by the field cancerization hypothesis [25].

Preoperative positive urine cytology is thought to be closely correlated to intraluminal seeding and implanting [26], which can effectively be eliminated by intravesical chemotherapy. Thus, it is reasonable that patients with preoperative positive urine cytology are sensitive to intravesical chemotherapy for the prevention of bladder tumor recurrence.

Aberrant EGFR signaling plays an important role in the tumorigenesis, migration, apoptosis resistance and stromal invasion of bladder cancer [27,28]. EGFR overexpression has been shown to be associated with high tumor grade, metastasis, poor prognosis, metaplastic morphology and secondary bladder recurrence in UTUC [9,10], and tumors with metaplastic features have been shown to react poorly to systemic chemotherapy [29,30]. Our study found that recurring bladder tumors were more likely to express higher levels of EGFR in patients that received intravesical chemotherapy than in those who did not. Additionally, compared with primary UTUC, recurrent bladder tumors expressed higher levels of EGFR. Thus, EGFR-overexpressing tumor cells, which are more inclined to have a metaplastic morphology, may also be insensitive to the instillation chemotherapy. As such, both intraluminal tumor cells and co-existing microscopic lesions overexpressing EGFR are more likely to survive instillation therapy and establish new bladder tumors.

Ki-67 is a nuclear protein that is indicative of cell proliferation. Similar to EGFR, Ki-67 overexpression is also suggestive of high tumor grade and poor prognosis [12,13]. In combination with CK20 overexpression, Ki-67 overexpression can serve as a predictor of pT1 urothelial bladder cancer recurrence [12]. Regarding UTUC, a previous study and this study show that low-level Ki-67 expression was an independent predictor of bladder tumor recurrence in patients without instillation therapy [13]. Our study also found that patients with low-level Ki-67 appeared to be more sensitive to intravesical chemotherapy; we also found that the subsequently recurring bladder tumors in patients with intravesical chemotherapy expressed more Ki-67 than those in the patients without intravesical chemotherapy. Interestingly, patients with Ki-67 overexpression appeared to be insensitive to intravesical chemotherapy. One possible explanation is that the high Ki-67 overexpression represents a high proliferation of dispersed intraluminal cancer cells, which provides an increased chance for these highly proliferative cancer cells to survive when exposed to chemotherapy drugs compared with other not highly proliferative cells. In this case, it would be less likely for these low-level Ki-67 cells (not highly proliferative cells) to seed and implant in the bladder wall and go on to develop secondary bladder cancer after intravesical chemotherapy.

Many conventional clinicopathological factors, such as ureteral tumor location, tumor multifocality, and preoperative positive urine cytology, have been recognized in numerous previous studies as risk factors for bladder cancer recurrence after RNU [5]. However, with the exception of preoperative positive urine cytology, which may be more closely related to intraluminal seeding and be more sensitive to intravesical chemotherapy, these clinicopathological factors cannot reliably indicate who will be sensitive to intravesical chemotherapy. Therefore, conventional clinicopathology alone cannot accurately be used to guide the clinical decision-making process with regard to postoperative intravesical instillations. However, Ki-67 and EGFR are promising biomarkers that can provide more reliable and accurate evidence to facilitate the identification of patients who will benefit from intravesical chemotherapy and thereby improve the implementation of individualized treatment strategies.

There are several limitations to this study. First, it was a single-center, retrospective study. Second, tumor distribution may vary in the tissue cores used for constructing the TMAs. This could lead to variability in the number of tumor-free spots in deeper sections, resulting in a decreased number of analyzable spots. Despite these limitations, our research identifies valuable biomarkers for the identification of not only patients who are at risk for bladder tumor recurrence but also those who would benefit the most from prophylactic, postoperative intravesical instillation therapy.

## Conclusions

Patients with low-level Ki-67 staining, negative EGFR staining and preoperative positive urine cytology appeared to be more sensitive to intravesical postoperative instillation therapy for the prevention of bladder recurrence after RNU for primary UTUC.

## Supporting Information

S1 Fig Differences in the incidence rates of bladder recurrence between patients with and without certain conventional clinicopathological factors before or after intravesical chemotherapy. The regression coefficients of the decreased recurrence rate after intravesical chemotherapy were not significantly different in patients with multiple or solitary tumors (a, P = 0.60) and with ureteric or pelvic tumor locations (b, P = 0.58). Furthermore, no interactions were observed between these clinicopathological factors and intravesical chemotherapy.
(TIF)

S1 Table Multivariate competing risk regression analyses for bladder tumor recurrence after radical nephroureterectomy in three sub-groups. (DOCX)

S2 Table Univariate and multivariate competing risk regression analyses for bladder tumor recurrence after radical nephroureterectomy in groups 1 and 2. (DOCX)
