# Supplementary figures and images for: Epidermal Growth Factor Receptor and Ki-67 as Predictive Biomarkers Identify Patients Who Will Be More Sensitive to Intravesical Instillations for the Prevention of Bladder Cancer Recurrence after Radical Nephroureterectomy
Source: PLoS One. 2016 Nov 21;11(11):e0166884. doi: 10.1371/journal.pone.0166884 (PMC5117727; doi:10.1371/journal.pone.0166884)

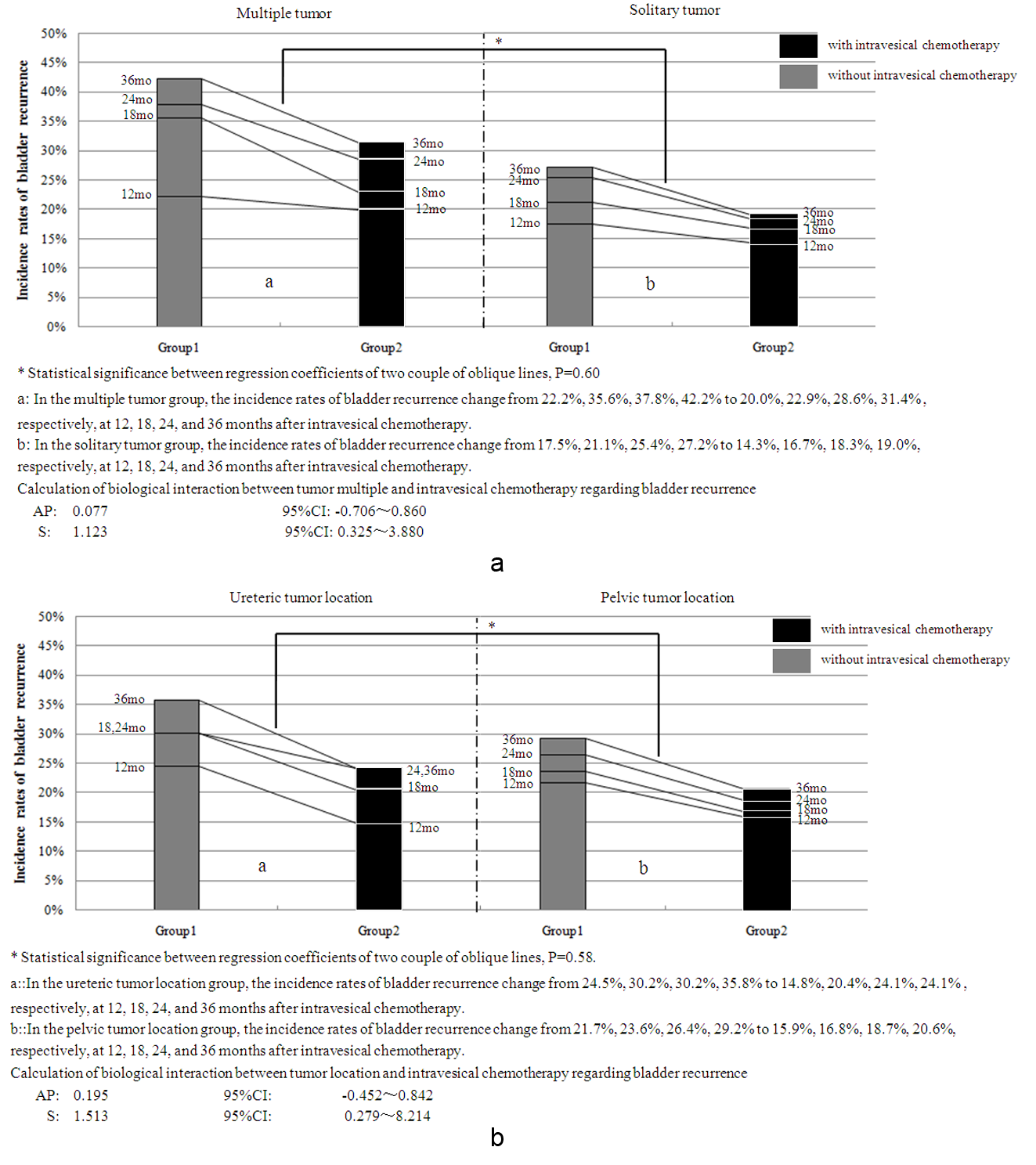

Supplement: S1 Fig — The regression coefficients of the decreased recurrence rate after intravesical chemotherapy were not significantly different in patients with multiple or solitary tumors (a, P = 0.60) and with ureteric or pelvic tumor locations (b, P = 0.58). Furthermore, no interactions were observed between these clinicopathological factors and intravesical chemotherapy. (TIF) [file pone.0166884.s001.tif]
