# Supplementary material for: Epidermal Growth Factor Receptor and Ki-67 as Predictive Biomarkers Identify Patients Who Will Be More Sensitive to Intravesical Instillations for the Prevention of Bladder Cancer Recurrence after Radical Nephroureterectomy
Source: PLoS One. 2016 Nov 21;11(11):e0166884. doi: 10.1371/journal.pone.0166884 (PMC5117727; doi:10.1371/journal.pone.0166884)
Supplement: S1 Table — (DOCX) [file pone.0166884.s002.docx]

| **Supplementary Table S1 . Multivariate competing risk regression analyses for bladder tumor recurrence after radical nephroureterectomy in three sub-groups** | | | | | | | | | | | | | | | | | | |
| --- | --- | --- | --- | --- | --- | --- | --- | --- | --- | --- | --- | --- | --- | --- | --- | --- | --- | --- |
| Group | EGFR | | | | | | | | | Ki-67 | | | | | | | | |
|  | EGFR-negative | | | | EGFR-positive | | | | | high-level Ki-67 | | | | | low-level Ki-67 | | | |
|  | HR^‡^ | 95% CI^§^ | | P | HR^‡^ | | 95% CI^§^ | P | | HR^‡^ | 95% CI^§^ | | P | | HR^‡^ | 95% CI^§^ | | P |
| Intravesical chemotherapy(yes) | 0.171 | 0.0746～0.392 | | <0.001^*^ | 0.814 | | 0.448～1.48 | 0.50 | | 1.151 | 0.712～3.22 | | 0.28 | | 0.453 | 0.243～0.845 | | 0.013^*^ |
| Gender (female) | 1.402 | 0.695～2.825 | | 0.34 | 0.878 | | 0.468～1.644 | 0.68 | | 0.344 | 0.135～0.88 | | 0.026^*^ | | 1.599 | 0.903～2.834 | | 0.11 |
| Grade(grade 3) | 0.890 | 0.226～3.501 | | 0.87 | 0.498 | | 0.270～0.918 | 0.025^*^ | | 0.507 | 0.229～1.12 | | 0.093 | | 1.080 | 0.517～2.255 | | 0.84 |
| Pathological T stage (T2) | 0.626 | 0.2120～1.846 | | 0.40 | 1.389 | | 0.64～3.014 | 0.41 | | 0.962 | 0.407～2.27 | | 0.93 | | 0.607 | 0.291～1.265 | | 0.18 |
| Pathological T stage (T3 or more) | 0.618 | 0.210～1.819 | | 0.38 | 2.744 | | 0.64～3.014 | 0.0051^*^ | | 1.351 | 0.585～3.12 | | 0.48 | | 0.997 | 0.536～1.856 | | 0.99 |
| Management of distal ureter (OBCE) | 1.414 | 0.325～6.149 | | 0.64 | 1.976 | | 0.408～9.574 | 0.40 | | 1.675 | 0.465～6.03 | | 4.395 | | 4.395 | 0.532～36.30 | | 0.17 |
| Surgical approach(OBCE) | 0.829 | 0.353～1.946 | | 0.67 | 3.398 | | 1.378～8.379 | 0.0079^*^ | | 1.672 | 0.709～3.94 | | 0.24 | | 0.905 | 0.502～1.633 | | 0.74 |
| Adjuvant chemotherapies (yes) | 0.314 | 0.049～2.034 | | 0.22 | 0.547 | | 0.226～1.324 | 0.180 | | 0.7616 | 0.275～2.11 | | 0.60 | | 1.085 | 0.436～2.703 | | 0.86 |
| Preoperative positive urine cytology(yes) | 7.593 | 3.562～16.18 | | <0.001^*^ | 0.927 | | 0.537～1.602 | 0.79 | | 1.918 | 0.911～4.04 | | 0.086 | | 1.766 | 1.022～3.051 | | 0.041^*^ |
| Ki-67 (low-level) | 1.384 | 0.540～3.553 | | 0.50 | 0.977 | | 0.533～1.790 | 0.94 | | — | — | | — | | — | — | | — |
| Tumor location (ureter) | 0.624 | 0.196～1.994 | | 0.43 | 1.890 | | 0.987～3.619 | 0.055^*^ | | 2.822 | 1.364～5.84 | | 0.0051^*^ | | 0.578 | 0.287～1.162 | | 0.12 |
| EGFR (positive) | — | — | | — | — | | — | — | | 4.609 | 2.059～10.32 | | <0.001^*^ | | 2.013 | 1.176～3.446 | | 0.011^*^ |
| Multifocality (multiple) | 11.524 | 3.90～34.052 | | <0.001^*^ | 0.586 | | 0.228～1.194 | 0.140 | | 1.011 | 0.41～2.49 | | 0.98 | | 2.73 | 1.610～4.63 | | <0.001^*^ |
| Status of surgical margins(positive) | 10.952 | 3.157～37.99 | | <0.001^*^ | 1.058 | | 0.493～2.269 | 0.89 | | 15.206 | 5.561～41.58 | | <0.001^*^ | | 1.516 | 0.642～3.581 | | <0.34 |
| Group | | | Preoperative positive urine cytology | | | | | | | | | | | | | | | |
|  |  |  | Negative | | | | | | | | | Positive | | | | | | |
|  |  |  | HR^‡^ | | | 95% CI^§^ | | | P | | | HR^‡^ | | 95% CI^§^ | | | P | |
| Intravesical chemotherapy(yes) | | | 0.754 | | | 0.365～1.556 | | | 0.45 | | | 0.4180 | | 0.212～0.792 | | | 0.008^*^ | |
| Gender (female) | | | 1.220 | | | 0.519～2.522 | | | 0.59 | | | 1.168 | | 0.60～2.275 | | | 0.65 | |
| Grade(grade 3) | | | 0.334 | | | 0.139～0.798 | | | 0.014^*^ | | | 0.863 | | 0.406～1.835 | | | 0.7 | |
| Pathological T stage (T2) | | | 0.682 | | | 0.287～1.621 | | | 0.39 | | | 2.515 | | 0.951～6.648 | | | 0.063 | |
| Pathological T stage (T3 or more) | | | 0.996 | | | 0.445～2.232 | | | 0.99 | | | 3.244 | | 1.326～7.936 | | | 0.01^*^ | |
| Management of distal ureter (OBCE) | | | 0.699 | | | 0.232～2.107 | | | 0.53 | | | 4.364 | | 0.601～31.696 | | | 0.15 | |
| Surgical approach(OBCE) | | | 0.675 | | | 0.295～1.546 | | | 0.35 | | | 3.716 | | 1.217～11.341 | | | 0.021^*^ | |
| Adjuvant chemotherapies (yes) | | | 1.147 | | | 0.441～2.984 | | | 0.78 | | | 0.536 | | 0.158～1.818 | | | 0.32 | |
| Preoperative positive urine cytology(yes) | | | — | | | — | | | — | | | — | | — | | | — | |
| Ki-67 (low-level) | | | 0.586 | | | 0.279～1.233 | | | 0.16 | | | 1.040 | | 0.531～2.034 | | | 0.91 | |
| Tumor location (ureter) | | | 1.790 | | | 0.778～4.118 | | | 0.17 | | | 0.793 | | 0.403～1.559 | | | 0.50 | |
| EGFR (positive) | | | 6.832 | | | 3.435～13.589 | | | <0.001^*^ | | | 1.936 | | 1.024～3.660 | | | 0.042^*^ | |
| Multifocality (multiple) | | | 1.055 | | | 0.462～2.413 | | | 0.90 | | | 2.175 | | 1.166～4.055 | | | 0.015^*^ | |
| Status of surgical margins(positive) | | | 3.304 | | | 1.456～7.497 | | | 0.0042^*^ | | | 1.583 | | 0.437～5.732 | | | 0.48 | |

† OBCE: Open bladder cuff excision

‡ HR: hazard ratio

§ 95% CI: 95% confidence interval

* Statistically significant at P < 0.05.
