# Supplementary material for: Epidermal Growth Factor Receptor and Ki-67 as Predictive Biomarkers Identify Patients Who Will Be More Sensitive to Intravesical Instillations for the Prevention of Bladder Cancer Recurrence after Radical Nephroureterectomy
Source: PLoS One. 2016 Nov 21;11(11):e0166884. doi: 10.1371/journal.pone.0166884 (PMC5117727; doi:10.1371/journal.pone.0166884)
Supplement: S2 Table — (DOCX) [file pone.0166884.s003.docx]

| **Supplementary Table S2. Univariate and multivariate competing risk regression analyses for bladder tumor recurrence after radical nephroureterectomy in groups 1 and 2** | | | | | | | | | | | | |
| --- | --- | --- | --- | --- | --- | --- | --- | --- | --- | --- | --- | --- |
| Group | Group 1 | | | | | | Group 2 | | | | | |
|  | Univariate | | | Multivariate | | | Univariate | | | Multivariate | | |
|  | HR^‡^ | 95% CI^§^ | P | HR^‡^ | 95% CI^§^ | P | HR^‡^ | 95% CI^§^ | P | HR^‡^ | 95% CI^§^ | P |
| Gender (female) | 1.02 | 0.551～1.90 | 0.94 | 0.935 | 0.480～1.82 | 0.84 | 1.35 | 0.649～2.81 | 0.42 | 1.377 | 0.573～3.31 | 0.47 |
| Grade | 0.738 | 0.38～1.43 | 0.37 | 0.814 | 0.374～1.77 | 0.60 | 0.802 | 0.361～1.78 | 0.59 | 0.621 | 0.237～1.63 | 0.33 |
| Status of surgical margins(positive) | 2.36 | 0.839～6.62 | 0.10 | 2.385 | 0.564～10.09 | 0.24 | 3.26 | 1.34～7.96 | 0.0093^*^ | 2.851 | 1.113～7.31 | 0.029^*^ |
| Pathological T stage (T2) | 0.893 | 0.409～1.95 | 0.78 | 1.007 | 0.382～2.66 | 0.99 | 1.43 | 0.588～3.48 | 0.43 | 1.094 | 0.411～2.91 | 0.86 |
| Pathological T stage (T3 or more) | 1.594 | 0.809～3.14 | 0.18 | 1.748 | 0.741～4.28 | 0.22 | 1.63 | 0.703～3.80 | 0.25 | 1.696 | 0.679～4.24 | 0.26 |
| Management of distal ureter (OBCE) | 1.93 | 0.253～14.7 | 0.53 | 1.684 | 0.209～13.55 | 0.62 | 1.57 | 0.409～6.02 | 0.51 | 2.081 | 0.472～9.18 | 0.33 |
| Surgical approach | 1.5 | 0.798～2.8 | 0.21 | 1.492 | 0.723～3.08 | 0.28 | 2.03 | 0.463～8.91 | 0.35 | 2.894 | 0.639～12.71 | 0.17 |
| Adjuvant chemotherapies (yes) | 0.622 | 0.245～1.58 | 0.32 | 0.641 | 0.196～2.09 | 0.46 | 1.58 | 0.609～4.08 | 0.35 | 1.210 | 0.429～3.41 | 0.72 |
| Preoperative positive urine cytology | 2.46 | 1.39～4.35 | 0.0021^*^ | 2.173 | 1.084～4.35 | 0.029^*^ | 1.12 | 0.563～2.23 | 0.75 | 1.143 | 0.544～2.40 | 0.72 |
| Ki-67 (low-level) | 0.474 | 0.265～0.848 | 0.012^*^ | 0.499 | 0.249～1.00 | 0.05^*^ | 0.849 | 0.425～1.70 | 0.64 | 1.581 | 0.673～3.71 | 0.29 |
| Tumor location (ureter) | 1.05 | 0.577～1.93 | 0.86 | 1.023 | 0.492～2.13 | 0.95 | 0.944 | 0.444～2.01 | 0.88 | 0.967 | 0.400～2.33 | 0.94 |
| EGFR (positive) | 2.23 | 1.26～3.93 | 0.0058^*^ | 2.238 | 1.168～4.29 | 0.15 | 3.79 | 1.77～8.11 | <0.001^*^ | 3.966 | 1.607～9.79 | 0.0028^*^ |
| Multifocality (multiple) | 1.66 | 0.933～2.95 | 0.085 | 1.835 | 1.007～3.35 | 0.047^*^ | 2.42 | 1.19～4.9 | 0.014^*^ | 2.473 | 0.889～6.88 | 0.083 |

† OBCE: Open bladder cuff excision

‡ HR: hazard ratio

§ 95% CI: 95% confidence interval

* Statistically significant at P < 0.05
